# Novel hemostatic gel and powder as rescue agents for arterial bleeding related to lumen-apposing metal stent placement

**DOI:** 10.1055/a-2512-4496

**Published:** 2025-01-23

**Authors:** Butros Fakhoury, Mohanad Awadalla, Michael Talanian, Tanya Zeina, Erika Tsuchiyose, Nikola Natov, Erik Holzwanger

**Affiliations:** 1 72054 Virginia Commonwealth University Medical Center, Richmond, United States; 2 1859 Beth Israel Deaconess Medical Center, Boston, United States; 3 1867 Tufts Medical Center, Boston, United States; 4 12261 Tufts University School of Medicine, Boston, United States

Intraprocedural bleeding following endoscopic ultrasound (EUS)-guided placement of lumen-apposing metal stents (LAMSs) is not an infrequent complication. Significant arterial bleeding may require interventions such as coil embolization or surgical intervention 1 . Identifying and accessing the bleeding site can be particularly challenging when it is obscured by the stent. Recent advances in topical hemostatic agents, such as gels and powders, offer the benefits of broad targeted therapy 2 . We report a unique case detailing the successful management of active arterial bleeding from the gastroepiploic artery after balloon dilation of a LAMS using topical hemostatic gel and powder ( Video 1 ).

Endoscopic ultrasound-guided placement of lumen-apposing metal stent in a 39-year-old man with recurrent walled-off pancreatic necrosis, complicated by arterial bleeding, successfully managed with a combination of hemostatic gel and powder.Video 1

A 39-year-old man with a history of necrotizing pancreatitis and walled-off pancreatic necrotic collections presented with abdominal pain and recurrent walled-off pancreatic necrosis. A computed tomography (CT) angiogram revealed no evidence of a pseudoaneurysm. Under EUS guidance, the stomach wall and the cyst were punctured using an electrocautery-equipped LAMS stent (AXIOS; Boston Scientific, Marlborough, Massachusetts, USA) ( Fig. 1 ). A significant arterial bleed occurred from beneath the stent during dilation ( Fig. 2 ). Hemostasis was achieved using topical hemostatic gel (PuraStat; 3-D Matrix Europe SAS, Caluire-et-Cuire, France) and powder (EndoClot Plus, Inc., Santa Clara, California, USA) ( Fig. 3 , Fig. 4 , Fig. 5 ). The post-procedure CT angiogram showed no active bleeding, and the culprit was identified as the gastroepiploic artery. The patient remained stable and was discharged without requiring additional intervention.

**Fig. 1:**
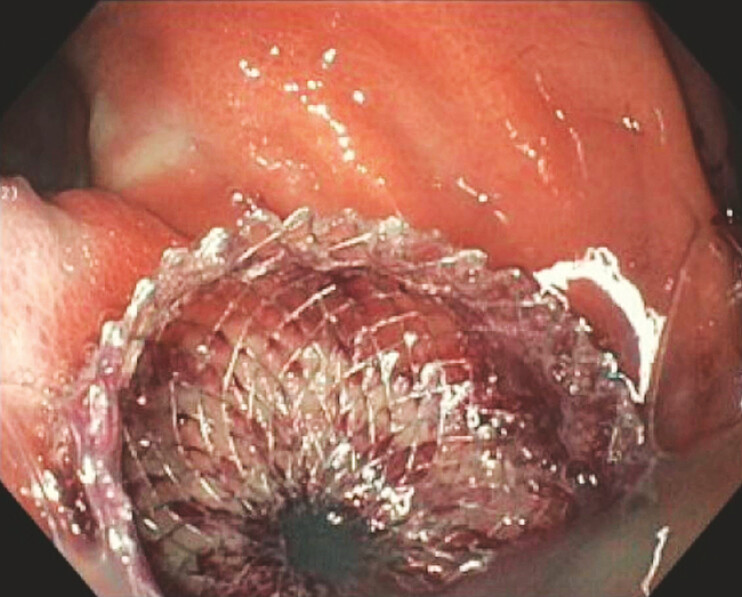
Successful deployment of the lumen-apposing metal stent (LAMS).

**Fig. 2:**
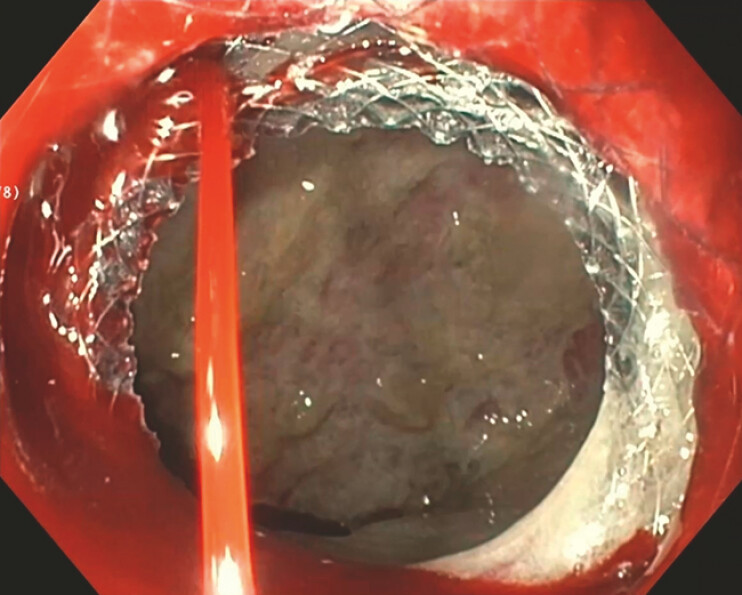
Brisk arterial bleeding beneath the LAMS stent post-dilation.

**Fig. 3:**
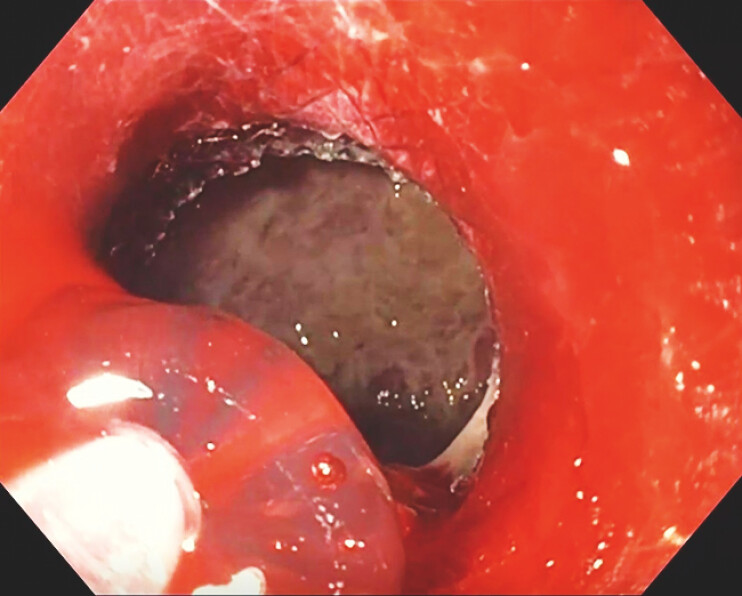
Administration of hemostatic gel at the suspected bleeding site using a specialized catheter.

**Fig. 4:**
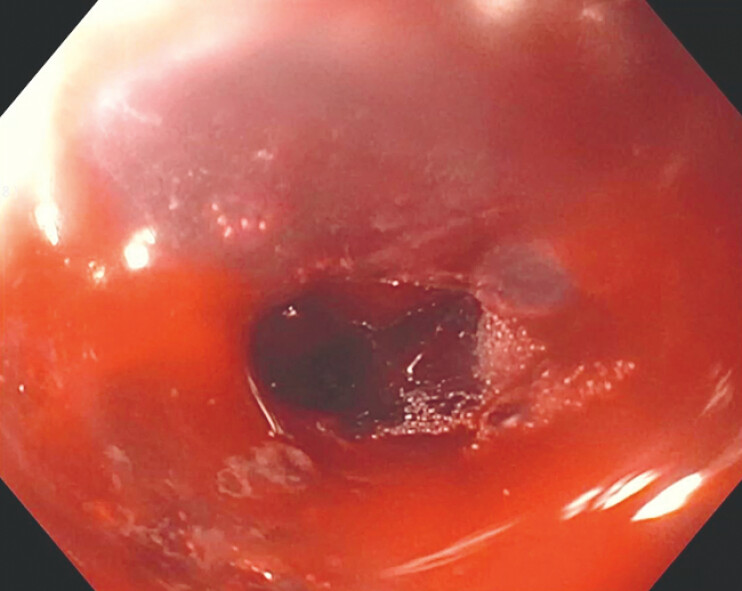
Mild residual oozing identified after the application of hemostatic gel.

**Fig. 5:**
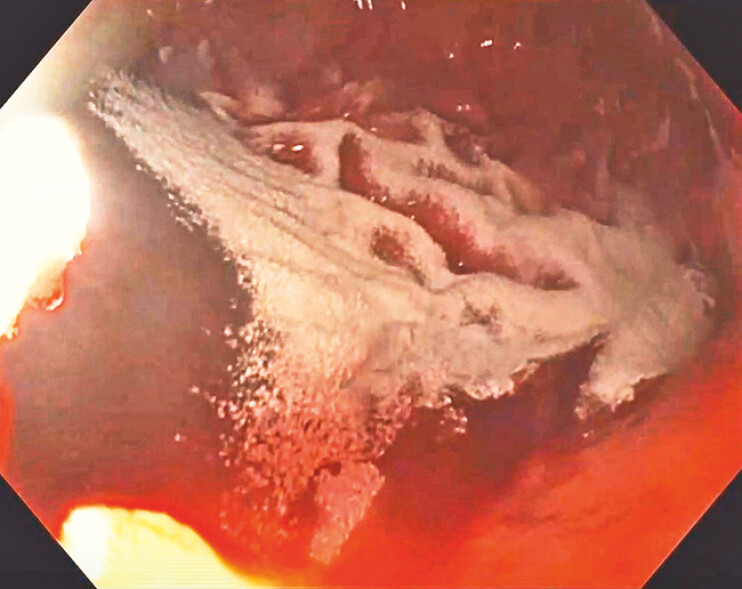
Endoscopic confirmation of hemostasis following the application of topical hemostatic agents.

Topical hemostatic agents achieve hemostasis by forming mechanical barriers that enhance clot formation 3 . Hemostatic gel, in particular, maintains visibility and has a lower rebleeding rate 4 . While these agents are approved by the U.S. Food and Drug Administration for venous bleeding, their efficacy in managing arterial bleeding remains uncertain 5 . This case demonstrates the evolving role of topical hemostatic agents in managing complex bleeding situations where traditional methods are impractical. Further studies are warranted to refine management strategies and evaluate the long-term outcomes.

Endoscopy_UCTN_Code_CPL_1AL_2AD

## Notes

Endoscopy E-Videos https://eref.thieme.de/e-videos
*E-Videos* is an open access online section of the journal *Endoscopy* , reporting on interesting cases and new techniques in gastroenterological endoscopy. All papers include a high-quality video and are published with a Creative Commons CC-BY license. Endoscopy E-Videos qualify for HINARI discounts and waivers and eligibility is automatically checked during the submission process. We grant 100% waivers to articles whose corresponding authors are based in Group A countries and 50% waivers to those who are based in Group B countries as classified by Research4Life (see: https://www.research4life.org/access/eligibility/ ). This section has its own submission website at https://mc.manuscriptcentral.com/e-videos .

## References

[1] Voermans RP Besselink MG Fockens P Endoscopic management of walled-off pancreatic necrosis J Hepatobiliary Pancreat Sci 2015 22 2026 10.1002/jhbp.180 25345777

[2] Kichler A Jang S Endoscopic hemostasis for non-variceal upper gastrointestinal bleeding: new frontiers Clin Endosc 2019 52 401 406 10.5946/ce.2018.103 31309768 PMC6785418

[3] Palmer R Braden B New and emerging endoscopic haemostasis techniques Frontline Gastroenterol 2015 6 147 152 10.1136/flgastro-2014-100540 28839802 PMC5369562

[4] de Nucci G Reati R Arena I Efficacy of a novel self-assembling peptide hemostatic gel as rescue therapy for refractory acute gastrointestinal bleeding Endoscopy 2020 52 773 779 10.1055/a-1145-3412 32316041

[5] Subramaniam S Kandiah K Thayalasekaran S Haemostasis and prevention of bleeding related to ER: The role of a novel self-assembling peptide United European Gastroenterol J 2019 7 155 162 10.1177/2050640618811504 PMC6374844 30788128

